# The associations between dietary flavonoid intake and the prevalence of diabetes mellitus: Data from the National Health and Nutrition Examination Survey 2007-2010 and 2017-2018

**DOI:** 10.3389/fendo.2023.1250410

**Published:** 2023-08-19

**Authors:** Yanjun Zhou, Peng Xu, Shaolei Qin, Yan Zhu, Ke Gu

**Affiliations:** ^1^ Department of Radiotherapy and Oncology, The Affiliated Hospital of Jiangnan University, Wuxi, Jiangsu, China; ^2^ Population Health Sciences, German Center for Neurodegenerative Diseases (DZNE), Bonn, Germany; ^3^ Wuxi Medical College, Jiangnan University, Wuxi, Jiangsu, China

**Keywords:** flavonoids, flavan 3-ols, catechins, diabetes mellitus, plant-based diets

## Abstract

**Background:**

Diabetes mellitus (DM) is a prominent health concern worldwide, leading to the high incidence of disability and mortality and bringing in heavy healthcare and social burden. Plant-based diets are reported associated with a reduction of DM risk. Plant-based diets are rich in flavonoids, which possess properties such as scavenging free radicals and exerting both anti-inflammatory and antioxidant effects.

**Purpose:**

However, whether dietary flavonoids are associated with the prevalence of DM remains controversial. The potential reasons for contradictory epidemiological outcomes on the association between dietary flavonoids and DM prevalence have not been determined.

**Methods:**

To address these limitations, we employed data from 22,481 participants in the National Health and Nutrition Examination Survey to explore the association between the intake of flavonoids and DM prevalence by weighted Logistic regression and weighted restricted cubic splines.

**Results:**

We found that the prevalence of DM was inversely associated with the intake of total flavonoids in the second quartile [Odds Ratio (OR) 0.78 95% confidence interval (CI) (0.63, 0.97), *p* = 0.028], in the third quartile [0.76 (0.60, 0.97), *p* = 0.031], and in the fourth quartile [0.80 (0.65, 0.97), *p* = 0.027]. However, the *p* for trend was not significant [0.94 (0.88, 1.01), p = 0.096]. Moreover, the association between DM prevalence and the intake of total flavonoids was significantly influenced by race (*p* for interaction = 0.006). In Mexican Americans, there was a significant positive association between DM prevalence and total flavonoid intake within the third quartile [1.04 (1.02, 1.07), *p* = 0.003]. Total flavan-3-ol and subtotal catechin intake exhibited a non-linear U-shaped association with DM prevalence (*p* for non-linearity < 0.0001 and *p* for non-linearity < 0.0001, respectively). Compared to the first quartile of corresponding intakes, consumption within the third quartile of subtotal catechins [0.70 (0.55, 0.89), *p* = 0.005] and total flavan-3-ols [0.65 (0.50, 0.84), *p* = 0.002] was associated with a lower prevalence of DM.

**Conclusion:**

Taken together, our study may provide preliminary research evidence for personalized improvement of dietary habits to reduce the prevalence of diabetes.

## 1 Introduction

Diabetes mellitus (DM) is a prevailing global health challenge associated with early death and disability, bringing in heavy health care and social burden (1). DM is a group of hyperglycemia disorders, including type 1 diabetes (T1D), type 2 diabetes (T2D), and gestational diabetes (2). T1D is considered to be an autoimmune disease, whereas T2D is mainly caused by defective insulin production and insulin resistance. The global prevalence of T2D is estimated to surge to reach 592 million by 2035 (3). In addition to the declining labor productivity caused by T2D, it is reported that more than 2 million deaths every year are associated with the neurological and vascular complications of T2D (4). Moreover, the amount of direct medical expenditure caused by T2D has increased from US$232 billion in 2007 to US$966 billion in 2021 (5). Therefore, the primary prevention of T2D has priority, which can improve the well-being of individuals and mitigate the social and economic burdens (6).

The risk factors of T2D include nonmodifiable factors, such as age and genetic variation, and modifiable factors, such as dietary patterns and increased physical activity (PA) (6). Among the changeable lifestyles, high-quality plant-based diets are reported to be associated with a lower risk of T2D (7–9). High-quality plant-based diets, namely fruits, vegetables, nuts, beans, and whole grains, rich in cellulose, flavonoids, and unsaturated fatty acids, demonstrate beneficial effects on T2D by improving insulin sensitivity, systemic inflammation, and weight maintenance (9).

Flavonoids, featured by a C6–C3–C6 structural backbone, are metabolites from plants. Flavonoids are found in fruits, vegetables, and other plant-based beverages and foods, have been proposed as anti-diabetic mediators (10). Flavonoids can be classified into six primary subclasses based on their chemical structure, which include anthocyanidins, flavan-3-ols, flavanones, flavones, flavonols, and isoflavones. Besides the antioxidant properties, dietary flavonoids can inhibit adipogenesis, improve insulin secretion and action, and alleviate inflammation, which may suggest protective effects during the development of DM (11, 12). However, the results from epidemiologic studies on the association between dietary flavonoids and the prevalence of DM are to date controversial. Some studies did not show the inverse association between DM risk and intake of flavonoids (13–16). Certain analyses revealed that flavonoid intake was associated with a lower risk of DM (17, 18). Furthermore, potential mechanisms that lead to inconsistent results remain to be elucidated.

Therefore, using all publicly available data from the USDA Food Code Flavonoid Values Database (the Flavonoid Database for short, 2007-2010 and 2017-2018), as well as flavonoid intake data from the Dietary Facts in America (WWEIA), National Health and Nutrition Examination Survey (NHANES), we explored the relationship between dietary flavonoid intake and the prevalence of diabetes (19). The impact of dietary flavonoid intake on the prevalence of diabetes was examined utilizing weighted logistic regression models and weighted restricted cubic spline (RCS) models. Stratified analyses were performed to identify subgroup effects, explore interaction effects and control for confounding factors. Moreover, the sensitivity analysis was conducted by excluding participants with impaired fasting glycaemia (IFG) and impaired glucose tolerance (IGT) from the population without DM using weighted Logistic regression models. Our findings will provide new insights into dietary flavonoid intakes in the prevention and management of DM in ethnically diverse populations.

## 2 Materials and methods

### 2.1 Study population

The NHANES is a comprehensive and nationally representative cross-sectional study that has been periodically conducted since the 1960s with the objective of evaluating the health and nutrition status of children and adults in the United States. The sampled population is recruited using a complex and multistage stratified sampling design, ensuring the national representativeness of study population (20). The NHANES was conducted in accordance with the Declaration of Helsinki, and approved by the Institutional Review Board (or Ethics Committee) of the institutional review board of the National Center for Health Statistics, CDC (protocol #2005-06, #2011-17, #2018-01).

**Figure 1:**
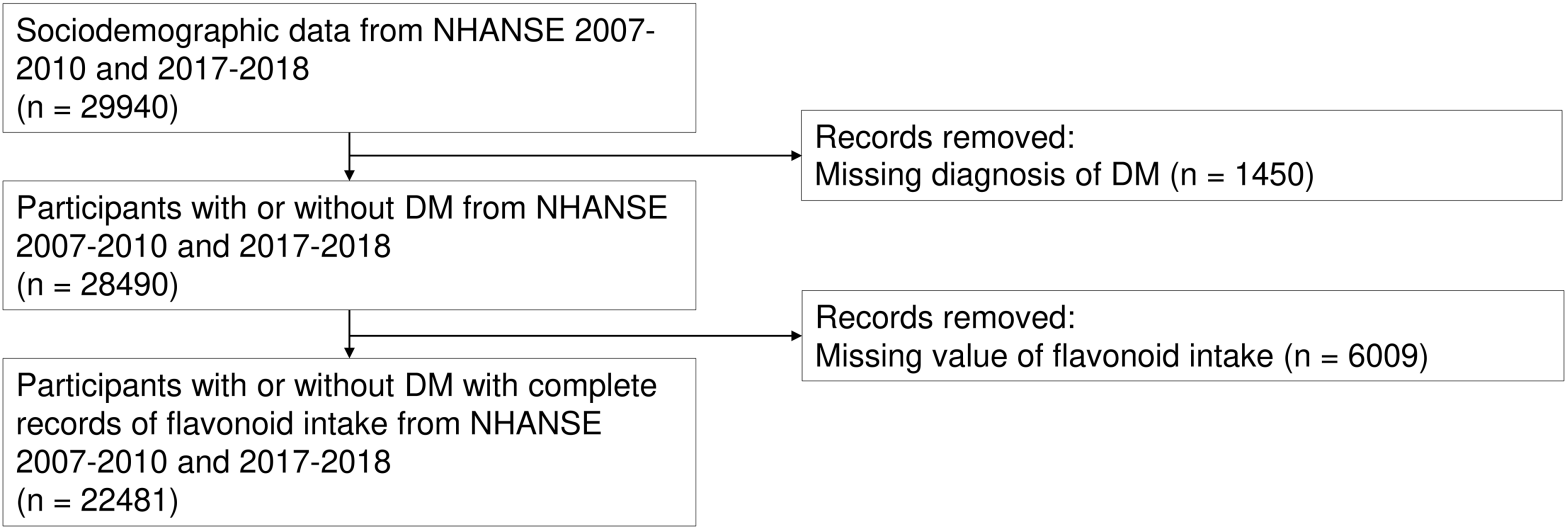
Flowchart of this study.

We acquired epidemiological data for 29,940 individuals aged 18 years or older who participated in the continuous NHANES cycles spanning from 2007 to 2010 and 2017 to 2018. Then, participants without information on the DM status (n = 1,450) and without complete records of dietary flavonoid intake assessments (n = 6,009) were excluded. A total of 22,481 participants were included in this study (**Figure 1**).

### 2.2 Assessment of flavonoid intakes

The values of dietary flavonoid intake were accessed from the flavonoid database, which was briefly described in our previous paper (21). Intakes of total flavonoids and primary subclasses of flavonoids were determined as the mean of each flavonoid's two-day intake based on the dietary recall. Consequently, we utilized the weight 'wtdr2d' in weighted analysis for participants who completed dietary recall on two days. The primary flavonoid subclasses are as follows: 1) total anthocyanidins (cyanidin, delphinidin, malvidin, pelargonidin, peonidin, and petunidin); 2) subtotal catechins ( (–)-epicatechin (–),-epicatechin 3-gallate (–),-epigallocatechin (–),-epigallocatechin 3-gallate, (+)-catechin, and (+)-gallocatechin); 3) total flavan-3-ol (subtotal catechins, theaflavin, theaflavin-3,3’-digallate, theaflavin-3’-gallate, theaflavin-3-gallate, and thearubigins); 4) total flavanones (eriodictyol, hesperetin, and naringenin); 5) total flavones (apigenin and luteolin); 6) total flavonols (isohamnetin, kaempferol, myricetin, and quercetin); 7) total isoflavones (diadzein, genistein, and glycitein).

### 2.3 Assessment of DM

The diagnostic criteria for DM include a physician's diagnosis of diabetes, glycohemoglobin HbA1c levels > 6.5%, fasting glucose levels ≧ 7.0 mmol/l, random blood glucose levels ≧ 11.1 mmol/l, two-hour blood glucose levels in the oral glucose tolerance test (OGTT2) ≧ 11.1 mmol/l, and utilization of antidiabetic agents or insulin therapy. The diagnostic criteria for IFG include a physician's diagnosis of IFG and fasting glucose ranging from 5.6 to 7.0 mmol/l. The diagnostic criteria for IGT include a physician's diagnosis of IGT and OGTT2 ranging from 7.8 to 11.0 mmol/l.

### 2.4 Assessment of covariates

The assessment of covariates was as previously described (21). In short, we obtained demographic and health-related data, including age, sex, ethnic background, educational attainment, marital condition, poverty income ratio (PIR), smoking habits, alcohol consumption, total duration, and total metabolic equivalent (MET) of physical activity (PA) per week, by administering questionnaires during the initial interview. Educational attainment was classified into three groups: less than 9 years, 9 to 12 years, and greater than 12 years. Marital condition was categorized as either partnered or unpartnered. BMI (body mass index) was computed by dividing weight in kilograms by the square of height in meters. Smoking habits were stratified into three groups: 1) never smoked, defined as consuming less than 100 cigarettes throughout one's lifetime; 2) former smoker, characterized by smoking more than 100 cigarettes throughout one's lifetime; and 3) current smoker, defined as having smoked more than 100 cigarettes throughout one's lifetime and currently smoking either some days or every day. Consumption of alcohol was classified as in (22). The healthy eating index (HEI) for the 2015 Edition was computed by summing the dietary intake over a period of two days (23). The dietary inflammatory index (DII) was computed as follows (24).

Hyperlipidaemia was identified when any of the subsequent criteria were met: triglycerides ≧ 150 mg/dL, low-density lipoprotein ≧ 130 mg/dL, high-density lipoprotein < 140 ng/dL, or utilization of antihyperlipidemic agents. A history of stroke or heart attack was considered indicative of cardiovascular disease. Chronic obstructive pulmonary disease (COPD) was defined as meeting any of the subsequent criteria: a forced expiratory volume at first second/forced vital capacity (FEV1/FVC) value < 0.7 after bronchodilator administration, a physician's diagnosis of emphysema, or utilization of COPD medications such as selective phosphodiesterase-4 inhibitors, mast cell stabilizers, leukotriene modifiers, and inhaled corticosteroids. Asthma was diagnosed upon fulfilment of any of the following criteria: a history of asthma diagnosis, occurrence of an asthma attack, or use of selective phosphodiesterase-4 inhibitors, mast cell stabilizers, leukotriene modifiers, and inhaled corticosteroids. A previous stroke is considered to be a history of stroke. Previous diagnosis of any type of cancer recognized as cancer history. The average blood pressure was computed as described by (25), and hypertension was diagnosed if any of the following conditions were met: systolic pressure ≧ 140 mmHg or diastolic pressure ≧ 90 mmHg on three separate occasions.

### 2.5 Statistical analysis

All statistical analyses were performed using R software (version 4.1.3). Based on the complex sampling design of NHANES, the SDMVPSU and SDMVSTRA procedures were employed to derive nationally representative estimates. The packages "NHANESR" and "survey" in R were utilized for data cleaning and statistical analyses. The "mice" package in R was utilized to impute missing covariate values. In the analysis of baseline information, continuous variables were presented as weighted mean ± standard deviation using one-way ANOVA to compare differences between groups; categorical variables were expressed as frequencies and percentages and compared using the Chi-Squared test. Then, four weighted logistic regression models were employed to examine the relationship between flavonoid consumption and the prevalence of DM. Crude model was unadjusted. Model 1 was adjusted by age, race, BMI, and daily energy intake (kcal). Model 2 was adjusted by age, race, BMI, and daily energy intake (kcal), total time of PA, smoking status, alcohol consumption, total score of HEI 2015 Edition, hypertension history, and hyperlipidemia history. Model 3 was adjusted by age, sex, race, BMI, and daily energy intake (kcal), total time of PA, smoking status, alcohol consumption, total score of HEI 2015 Edition, hypertension history, and hyperlipidemia history. To investigate possible nonlinear associations, we utilized weighted RCS implemented in the "rms" package. As subgroup weighted Logistic analyses, effects of flavonoid intake on prevalence of DM were stratified by age, sex, race, BMI, daily energy intake, total time of PA, smoking habits, alcohol intake, and history of hypertension or hyperlipidaemia. RCS analysis was employed to examine the correlation between flavonoid intake and DM occurrence across different races. Weighted Logistic regression was performed to compute odds ratio (OR) and corresponding 95% confidence intervals (CIs). A significance level of *p* < 0.05 was adopted as the threshold for statistical significance. In the weighted logistic regression models, a CI that did not include the value of 1 was deemed to indicate statistical significance.

## 3 Results

### 3.1 Characteristics of participants

A total of 22,481 adult subjects (aged 18 years or over) in the NHANSE (2007-2010 and 2017-2018) were included in this study, which was representative of the 30,467,458 US non-hospitalized population. Baseline characteristics for participants are presented according to the status of DM in **Table 1**. A total of 2764 cases of DM were observed in our study. The mean age of participants was 34.94 years among those without DM, and 59.04 years among those with DM (**Table 1**, *p* < 0.0001). The participants with DM have a lower proportion with more than 12 years of education (50.56%) and more frequency with partners (63.12%), compared to the participants without DM. Although with more heavy consumption of alcohol (23.22%), higher daily energy intake (4064.44 kcal), and lower total score of HEI (52.06), participants without DM exhibited a health-conscious lifestyle pattern with lower BMI (26.11 kg/m^2^), more physical activity (1290.11 min/week and 5216.20 MET/week), higher frequency of never-smoking (57.85%), compared to the participants with DM (**Table 1**). No significant differences were observed in terms of sex, DII, and PIR between healthy participants and participants with DM (**Table 1**). Furthermore, individuals diagnosed with DM exhibited a heightened occurrence of hyperlipidemia (87.48%), CVD (25.97%), asthma and/or COPD (21.32%), stroke (9.25%), cancer (17.56%), and hypertension (69.79%), compared to those without DM (**Table 1**). Furthermore, a lower intake of daidzein, genistein, glycitein, hesperetin, and total isoflavones was observed in the participants with DM (**Table 1**). However, it was observed that a higher intake of luteolin, isorhamnetin, myricetin, quercetin, total flavan 3-ols, total flavones, total flavonols, and total flavonoids in the participants with DM (**Table 1**).

**Table 1:** Characteristics of participants in the cohorts according to DM status.

Variable	Participants without DM	Participants with DM	*p*-value
Cases (n)	19717	2764	
Sociodemographic, lifestyle, and health-related variables			
Age (years)	34.94 (0.31)	59.04 (0.56)	< 0.0001
Sex			0.39
Female	10112 (52.09%)	1368 (50.40%)	
Male	9605 (47.91%)	1396 (49.60%)	
Race			0.03
Non-Hispanic Black	4081 (11.78%)	690 (14.42%)	
Mexican American	3810 (10.47%)	481 (9.30%)	
Other races	1701 (7.60%)	207 (7.36%)	
Non-Hispanic White	10125 (70.14%)	1386 (68.92%)	
Education			< 0.0001
<9 years	1606 (4.33%)	452 (9.88%)	
9-12 years	7792 (35.97%)	1132 (39.56%)	
>12 years	10319 (59.70%)	1180 (50.56%)	
Marital status			< 0.0001
Without partner	10590 (47.68%)	1150 (36.88%)	
With partner	9127 (52.32%)	1614 (63.12%)	
Smoking status			< 0.0001
Former	2581 (22.61%)	936 (35.70%)	
Never	6421 (57.85%)	1333 (48.70%)	
Now	2354 (19.54%)	433 (15.60%)	
Alcohol usage			< 0.0001
Former	1272 (9.84%)	565 (19.49%)	
Heavy	2223 (23.22%)	289 (13.01%)	
Mild	3592 (38.25%)	796 (38.40%)	
Moderate	1726 (18.59%)	266 (14.65%)	
Never	1317 (10.10%)	398 (14.45%)	
BMI (kg/m^2^)	26.11 (0.09)	33.28 (0.28)	< 0.0001
Total score of HEI 2015	52.06 (0.32)	54.31 (0.48)	< 0.0001
DII	1.67 (0.04)	1.70 (0.06)	0.69
Total time of PA (mins/week)	1290.11 (27.20)	1018.46 (52.39)	< 0.0001
Total MET of PA (/week)	5216.20 (140.38)	3504.16 (214.12)	< 0.0001
Daily energy intake (kcal)	4064.44 (21.56)	3759.70 (59.83)	< 0.0001
PIR	2.88 (0.04)	2.90 (0.06)	0.85
Hyperlipidemia			< 0.0001
No	7413 (42.17%)	381 (12.52%)	
Yes	9199 (57.83%)	2375 (87.48%)	
CVD			< 0.0001
No	10208 (93.99%)	1988 (74.03%)	
Yes	952 (6.01%)	708 (25.97%)	
COPD and asthma			< 0.0001
COPD and asthma	231 (1.82%)	121 (4.18%)	
Asthma	1345 (11.76%)	336 (12.95%)	
COPD	357 (2.86%)	127 (4.19%)	
No	9463 (83.56%)	2116 (78.68%)	
Stroke			< 0.0001
No	10783 (97.66%)	2427 (90.75%)	
Yes	365 (2.34%)	261 (9.25%)	
Cancer			< 0.0001
No	10115 (91.42%)	2267 (82.44%)	
Yes	1037 (8.58%)	425 (17.56%)	
Hypertension			< 0.0001
No	15519 (77.36%)	777 (30.21%)	
Yes	4198 (22.64%)	1987 (69.79%)	
Dietary intake of flavonoids (mg/day)			
Daidzein	0.71 (0.04)	0.51 (0.09)	0.03
Genistein	1.00 (0.06)	0.69 (0.13)	0.02
Glycitein	0.14 (0.01)	0.10 (0.02)	0.02
Cyanidin	2.42 (0.15)	2.68 (0.30)	0.41
Petunidin	1.04 (0.08)	1.16 (0.14)	0.4
Delphinidin	1.49 (0.12)	1.56 (0.16)	0.67
Malvidin	4.45 (0.26)	4.32 (0.42)	0.73
Pelargonidin	1.72 (0.15)	1.32 (0.16)	0.08
Peonidin	1.77 (0.14)	1.89 (0.31)	0.73
Catechin	6.96 (0.15)	7.50 (0.52)	0.31
Epigallocatechin	12.94 (0.55)	18.65 (3.18)	0.08
Epicatechin	9.53 (0.20)	9.83 (0.86)	0.73
Epicatechin-3-gallate	8.35 (0.37)	11.93 (1.96)	0.07
Epigallocatechin-3-gallate	22.00 (0.99)	33.91 (7.49)	0.11
Theaflavin	1.26 (0.07)	1.52 (0.14)	0.08
Thearubigins	72.41 (3.79)	87.82 (7.42)	0.06
Eriodictyol	0.16 (0.01)	0.14 (0.02)	0.26
Hesperetin	9.04 (0.26)	7.75 (0.49)	0.01
Naringenin	3.16 (0.13)	3.44 (0.38)	0.44
Apigenin	0.19 (0.02)	0.21 (0.02)	0.47
Luteolin	0.61 (0.02)	0.71 (0.03)	0.01
Isorhamnetin	0.71 (0.02)	0.79 (0.02)	0.01
Kaempferol	3.82 (0.09)	4.21 (0.19)	0.06
Myricetin	1.25 (0.03)	1.55 (0.12)	0.02
Quercetin	9.84 (0.18)	11.02 (0.37)	0.01
Theaflavin-3,3’-digallate	1.39 (0.08)	1.67 (0.15)	0.09
Theaflavin-3’-gallate	1.18 (0.07)	1.42 (0.13)	0.09
Theaflavin 3-gallate	1.00 (0.06)	1.20 (0.11)	0.08
Gallocatechin	1.34 (0.06)	1.73 (0.19)	0.05
Subtotal Catechins	61.12 (2.22)	83.55 (14.09)	0.12
Total Isoflavones	1.85 (0.12)	1.29 (0.24)	0.02
Tota Anthocyanidins	12.89 (0.61)	12.93 (1.13)	0.97
Total Flavan 3-ols	138.36 (5.97)	177.19 (17.04)	0.03
Total Flavanones	12.36 (0.37)	11.32 (0.82)	0.18
Total Flavones	0.80 (0.03)	0.92 (0.04)	0.01
Total Flavonols	15.62 (0.30)	17.57 (0.66)	0.01
Total Sum of all 29 flavonoids	181.88 (6.39)	221.22 (17.73)	0.04

PIR, poverty income ratio; BMI, body mass index; DII, dietary inflammatory index; HEI, healthy eating index. Continuous normal variables were presented as weighted mean ± standard deviation and compared using One-way ANOVA. Categorical variables were presented as frequencies and percentages and compared using Chi-Squared test.

Furthermore, comparisons were made among races in regards to the characteristics of the study population (**Supplementary Table S1**). Compared to other racial groups, Mexican American had a lower mean age (p < 0.0001, **Supplementary Table S1**). Among the different racial groups, Mexican American had the lowest proportion of individuals with more than 12 years of education (*p* < 0.0001), the highest proportion with heavy alcohol consumption (*p* < 0.0001), the highest total MET of PA (*p* < 0.0001), lowest PIR (*p* < 0.0001), lowest prevalence of DM (*p* = 0.03), and the lowest intake of total flavonoids (*p* < 0.0001) (**Supplementary Table S1**). Additionally, a summary of the study population's characteristics based on quartiles of flavonoid intake can be found in **Supplementary Table S2**. Flavonoid intake varied across racial groups, with a higher proportion of Non-Hispanic White individuals having higher intake levels (*p* < 0.0001, **Supplementary Table S2**).

### 3.2 Associations between flavonoid intake and prevalence of DM

To further assess the potential association between flavonoid intake and risk of DM, the association was fully adjusted by age, sex, race, BMI, and daily energy intake, total time of PA, smoking status, alcohol consumption, total score of HEI 2015 Edition, hypertension history, and hyperlipidemia history in weighted Logistic regression. As more than 50% of the survey population reported no isoflavone intake, we categorized isoflavone consumption into two groups using median intake. The remaining flavonoid subclasses were classified into quartiles according to their intake levels. After full adjustment in **Table 2**, an inverse relationship was observed between total flavonoid intake and prevalence of DM within the second quartile [0.78 (0.63, 0.97), *p* = 0.028], in the third quartile [0.76 (0.60, 0.97), *p* = 0.031], and in the fourth quartile [0.80 (0.65, 0.97), *p* = 0.027], compared to that in the first quartile. However, the *p* for trend was not significant [0.94 (0.88, 1.01), *p* = 0.096]. Moreover, the prevalence of DM had an inverse relationship with the intake of total flavan 3-ols in the third quartile [0.65 [0.50, 0.84], *p* = 0.002], compared to that in the first quartile. Similarly, the prevalence of DM was negatively linked to the intake of subtotal catechins in third quartile [0.70 (0.55, 0.89), *p* = 0.005], compared to that in the first quartile (**Table 2**). These results may suggest a nonlinear association between the prevalence of DM and intake of total flavan 3-ols as well as subtotal catechins (**Table 2**).

**Table 2:** ORs (95% CIs) of DM prevalence according to flavonoid intake in the NHANSE (2007-2010 and 2017-2018).

Flavonoid intake	Q1	Q2		Q3		Q4		OR (95%CI)	*p* for trend
		OR (95%CI)	*p* Value	OR (95%CI)	*p* Value	OR (95%CI)	*p* Value		
Total Sum of all 29 flavonoids (mg/day)	≤20.645	20.645-49.735		49.735-140.145		>140.145			
Crude Model	Ref	0.80 (0.67, 0.96)	0.018	0.92 (0.76, 1.12)	0.392	1.07 (0.89, 1.27)	0.461	1.04 (0.98, 1.11)	0.198
Model 1	Ref	0.79 (0.63, 0.99)	0.038	0.76 (0.60, 0.96)	0.021	0.76 (0.62, 0.92)	0.008	0.92 (0.86, 0.99)	0.018
Model 2	Ref	0.77 (0.62, 0.95)	0.017	0.74 (0.58, 0.94)	0.017	0.76 (0.62, 0.93)	0.009	0.93 (0.87, 1.00)	0.039
Model 3	Ref	0.78 (0.63, 0.97)	0.028	0.76 (0.60, 0.97)	0.031	0.80 (0.65, 0.97)	0.027	0.94 (0.88, 1.01)	0.096
Total Flavan 3-ols (mg/day)	≤4.605	4.605-13.245		13.245-59.525		>59.525			
Crude Model	Ref	0.77 (0.62, 0.96)	0.023	0.64 (0.50, 0.81)	<0.001	1.04 (0.84, 1.27)	0.729	1.01 (0.94, 1.08)	0.764
Model 1	Ref	0.80 (0.62, 1.02)	0.068	0.64 (0.50, 0.83)	0.001	0.80 (0.63, 1.01)	0.057	0.93 (0.86, 1.00)	0.059
Model 2	Ref	0.78 (0.61, 1.00)	0.052	0.64 (0.49, 0.83)	0.001	0.80 (0.63, 1.02)	0.071	0.94 (0.87, 1.01)	0.102
Model 3	Ref	0.79 (0.61, 1.01)	0.058	0.65 (0.50, 0.84)	0.002	0.84 (0.66, 1.07)	0.146	0.95 (0.88, 1.03)	0.212
Subtotal Catechins (mg/day)	≤4.505	4.505-12.745		12.745-37.725		>37.725			
Crude Model	Ref	0.81 (0.66, 1.00)	0.048	0.68 (0.55, 0.83)	<0.001	0.99 (0.81, 1.20)	0.913	0.99 (0.93, 1.06)	0.812
Model 1	Ref	0.82 (0.64, 1.05)	0.113	0.70 (0.55, 0.88)	0.003	0.80 (0.64, 1.01)	0.058	0.93 (0.86, 1.00)	0.058
Model 2	Ref	0.81 (0.63, 1.03)	0.082	0.69 (0.54, 0.88)	0.004	0.80 (0.63, 1.01)	0.061	0.94 (0.87, 1.01)	0.082
Model 3	Ref	0.82 (0.64, 1.04)	0.094	0.70 (0.55, 0.89)	0.005	0.83 (0.66, 1.05)	0.116	0.95 (0.88, 1.02)	0.156
Total Isoflavones (mg/day)	≤0.010	0.010-0.065							
Crude Model	Ref	0.98 (0.86, 1.13)	0.812						
Model 1	Ref	0.99 (0.86, 1.14)	0.883						
Model 2	Ref	1.00 (0.85, 1.17)	0.989						
Model 3	Ref	1.01 (0.86, 1.19)	0.872						
Total Anthocyanidins (mg/day)	≤ 0.125	0.125-1.910		1.910-9.340		>9.340			
Crude Model	Ref	0.89 (0.72, 1.09)	0.254	1.08 (0.86, 1.35)	0.509	0.93 (0.74, 1.17)	0.539	1.00 (0.92, 1.07)	0.904
Model 1	Ref	0.85 (0.66, 1.09)	0.199	0.96 (0.75, 1.24)	0.748	0.77 (0.61, 0.97)	0.029	0.93 (0.86, 1.01)	0.075
Model 2	Ref	0.84 (0.65, 1.07)	0.146	0.96 (0.74, 1.23)	0.719	0.77 (0.60, 1.00)	0.046	0.94 (0.86, 1.02)	0.124
Model 3	Ref	0.86 (0.67, 1.11)	0.238	0.99 (0.77, 1.28)	0.95	0.82 (0.64, 1.05)	0.109	0.95 (0.88, 1.04)	0.251
Total Flavanones (mg/day)	≤ 0.020	0.020-0.660		0.660-17.730		>17.730			
Crude Model	Ref	1.25 (1.04, 1.51)	0.018	0.92 (0.75, 1.13)	0.424	1.04 (0.86, 1.26)	0.687	0.98 (0.92, 1.04)	0.446
Model 1	Ref	1.07 (0.88, 1.31)	0.47	0.88 (0.69, 1.11)	0.264	0.84 (0.68, 1.05)	0.117	0.93 (0.86, 0.99)	0.036
Model 2	Ref	1.08 (0.88, 1.32)	0.45	0.90 (0.69, 1.17)	0.402	0.85 (0.66, 1.09)	0.189	0.93 (0.86, 1.01)	0.093
Model 3	Ref	1.09 (0.89, 1.34)	0.371	0.94 (0.73, 1.21)	0.622	0.86 (0.68, 1.10)	0.223	0.94 (0.87, 1.02)	0.123
Total Flavones (mg/day)	≤0.110	0.110-0.345		0.345-0.845		>0.845			
Crude Model	Ref	1.31 (1.10, 1.56)	0.003	1.28 (1.04, 1.59)	0.023	1.56 (1.29, 1.88)	<0.001	1.14 (1.07, 1.21)	<0.001
Model 1	Ref	0.99 (0.82, 1.20)	0.93	0.93 (0.73, 1.19)	0.566	0.99 (0.84, 1.18)	0.942	1.00 (0.94, 1.06)	0.882
Model 2	Ref	0.99 (0.81, 1.21)	0.903	0.95 (0.74, 1.23)	0.71	1.02 (0.83, 1.27)	0.816	1.01 (0.94, 1.08)	0.833
Model 3	Ref	1.02 (0.83, 1.25)	0.851	1.00 (0.77, 1.30)	0.982	1.06 (0.86, 1.30)	0.575	1.02 (0.95, 1.09)	0.629
Total Flavonols	≤4.735	4.735-9.245		9.245-17.330		>17.330			
Crude Model	Ref	1.35 (1.14, 1.60)	0.001	1.57 (1.33, 1.86)	<0.001	1.48 (1.24, 1.77)	<0.001	1.12 (1.06, 1.19)	<0.001
Model 1	Ref	1.13 (0.87, 1.46)	0.352	1.02 (0.83, 1.25)	0.851	0.88 (0.72, 1.09)	0.234	0.94 (0.88, 1.00)	0.049
Model 2	Ref	1.15 (0.87, 1.51)	0.32	1.06 (0.85, 1.33)	0.572	0.90 (0.71, 1.14)	0.379	0.94 (0.88, 1.01)	0.095
Model 3	Ref	1.16 (0.89, 1.53)	0.266	1.09 (0.87, 1.36)	0.457	0.92 (0.73, 1.17)	0.478	0.95 (0.88, 1.02)	0.132

Crude model: unadjusted; Model 1: adjusted by age, race, BMI, and daily energy intake (kcal); Model 2: adjusted by age, race, BMI, and daily energy intake (kcal), total time of PA, smoking status, alcohol consumption, total score of HEI 2015 Edition, hypertension history, and hyperlipidemia history; Model 3: adjusted by age, sex, race, BMI, and daily energy intake (kcal), total time of PA, smoking status, alcohol consumption, total score of HEI 2015 Edition, hypertension history, and hyperlipidemia history. BMI, body mass index; HEI, healthy eating index; PA, physical activity.

**Figure 2:**
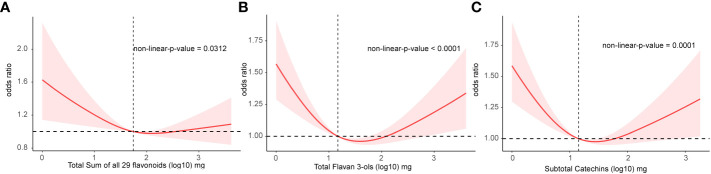
The association of flavonoid intake with prevalence of DM by restricted cubic splines. Y axis stands for the Odds ratio of DM, and X axis stands for the log10 transformed intake of total flavonoids **(A)**, total flavan 3-ols **(B)**, and subtotal catechins **(C)**. Models by restricted cubic splines were adjusted for age, sex, race, BMI, daily energy intake, total time of PA, smoking status, alcohol consumption, total score of HEI, hypertension history and hyperlipidaemia history.

As observed in **Table 2**, there might be non-linear associations between the intake of total flavan 3-ols as well as subtotal catechins, therefore, we employed RCS to explore the nonlinear associations (**Figure 2** and **Figure 1** There were significantly non-linear association between the prevalence of DM and the intake of total flavonoids, total flavan 3-ols, and subtotal catechins (*p* = 0.031, *p* < 0.0001, and *p* = 0.0001, respectively, **Figures 2A**) by RCS. Among the results above, the associations between the prevalence of DM and the intake of total flavan 3-ols and subtotal catechins demonstrated a U-shaped association. However, the associations between other flavonoid subclasses and the prevalence of DM did not reach a significance (**Supplementary Figure 1a-1e**).

### 3.3 Stratified analysis of the correlation between flavonoid intake and DM prevalence

To identify subgroup effects and explore interaction effects on the correlation between flavonoid intake and prevalence of DM, stratified analysis was conducted based on age, sex, race, BMI, daily energy intake, total time of PA, smoking habits, alcohol intake, and history of hypertension or hyperlipidemia and each stratification was fully adjusted by weighted Logistic regression (**Table 3**).

**Table 3:** Stratified association between DM prevalence and total flavonoid intake in the NHANSE (2007-2010 and 2017-2018).

Variables	Q1 (≤20.645)	Q2 (20.645-49.735)		Q3 (49.735-140.145)		Q4 (>=140.145)		*p* for interaction
	N = 5624	N = 5618		N = 5619		N = 5620		
		OR (95%CI)	*p* value	OR (95%CI)	*p* value	OR (95%CI)	*p* value	
Age (Years)								0.532
<50		0.99 (0.98, 1.01)	0.261	1.01 (0.99, 1.02)	0.373	1.00 (0.98, 1.01)	0.594	
>=50		0.96 (0.92, 1.00)	0.051	0.93 (0.89, 0.98)	0.007	0.95 (0.91, 0.99)	0.012	
Sex								0.702
Female		0.99 (0.97, 1.01)	0.424	1.00 (0.98, 1.02)	0.87	0.99 (0.97, 1.01)	0.345	
Male		0.99 (0.96, 1.01)	0.313	0.97 (0.95, 1.00)	0.083	0.96 (0.94, 0.99)	0.004	
Race								0.006
Non-Hispanic White		0.99 (0.96, 1.01)	0.325	0.99 (0.96, 1.01)	0.245	0.97 (0.95, 0.99)	0.012	
Non-Hispanic Black		1.00 (0.96, 1.03)	0.774	0.97 (0.94, 1.00)	0.078	0.97 (0.94, 1.01)	0.125	
Mexican American		1.02 (0.99, 1.05)	0.223	1.04 (1.02, 1.07)	0.003	1.04 (1.00, 1.09)	0.061	
Other Race		0.96 (0.91, 1.02)	0.147	0.96 (0.91, 1.03)	0.244	0.95 (0.89, 1.02)	0.152	
BMI (kg/m^2^)								0.247
<25.3		0.97 (0.93, 1.01)	0.105	0.99 (0.96, 1.04)	0.777	0.96 (0.93, 0.99)	0.013	
>=25.3		0.98 (0.95, 1.01)	0.117	0.98 (0.95, 1.01)	0.212	0.97 (0.95, 1.00)	0.031	
Daily energy intake (kcal)								0.159
<3584		0.99 (0.97, 1.02)	0.476	0.99 (0.97, 1.02)	0.554	0.98 (0.96, 1.01)	0.13	
>=3584		0.98 (0.96, 1.01)	0.155	0.98 (0.95, 1.00)	0.034	0.97 (0.94, 0.99)	0.005	
Total time of PA (mins/week)								0.313
<600		0.98 (0.95, 1.00)	0.089	0.98 (0.95, 1.01)	0.133	0.96 (0.93, 0.98)	0.001	
>=600		1.00 (0.98, 1.02)	0.988	1.00 (0.97, 1.02)	0.726	1.00 (0.98, 1.02)	0.829	
Smoking status								0.326
Never		1.00 (0.98, 1.02)	0.737	0.99 (0.96, 1.01)	0.274	0.97 (0.95, 1.00)	0.042	
Former		0.99 (0.94, 1.04)	0.769	0.98 (0.94, 1.02)	0.392	1.01 (0.96, 1.06)	0.789	
Current		0.97 (0.93, 1.01)	0.105	0.99 (0.96, 1.04)	0.777	0.96 (0.93, 0.99)	0.013	
Alcohol Consumption								0.352
Never		1.04 (0.98, 1.10)	0.17	1.01 (0.96, 1.06)	0.762	1.01 (0.96, 1.06)	0.66	
Former		0.98 (0.92, 1.05)	0.567	1.01 (0.94, 1.08)	0.86	0.93 (0.88, 0.98)	0.009	
Mild		0.96 (0.92, 1.01)	0.129	0.93 (0.89, 0.97)	0.004	0.95 (0.91, 0.99)	0.016	
Moderate		0.95 (0.90, 1.00)	0.066	1.02 (0.96, 1.08)	0.561	1.03 (0.97, 1.10)	0.312	
Heavy		0.98 (0.94, 1.02)	0.273	1.00 (0.94, 1.06)	0.917	0.98 (0.94, 1.02)	0.344	
Total score of HEI (2015 Edition)								0.556
<51.44		0.98 (0.96, 1.01)	0.214	1.00 (0.97, 1.02)	0.713	0.97 (0.95, 0.99)	0.01	
>=51.44		1.00 (0.97, 1.04)	0.795	1.00 (0.97, 1.03)	0.828	0.99 (0.97, 1.02)	0.722	
Hypertension								0.377
No		0.99 (0.98, 1.00)	0.136	1.00 (0.98, 1.01)	0.622	0.99 (0.97, 1.01)	0.262	
Yes		0.97 (0.92, 1.02)	0.172	0.94 (0.89, 1.00)	0.06	0.95 (0.90, 0.99)	0.014	
Hyperlipidemia								0.661
No	0.99 (0.97, 1.00)	0.02	0.99 (0.97, 1.01)	0.272	0.99 (0.97, 1.00)	0.097		
Yes	0.99 (0.96, 1.01)	0.397	0.98 (0.96, 1.01)	0.192	0.97 (0.95, 1.00)	0.048		

BMI, body mass index; HEI, healthy eating index; PA, physical activity.

Based on the findings presented in **Table 3**, race exhibited a significant influence on the association between DM prevalence and total flavonoid intake (*p* for interaction = 0.006). In non-Hispanic whites, an inverse correlation was observed between DM prevalence and flavonoid intake within the fourth quartile [0.97 (0.95, 0.99), *p* = 0.012] (**Table 3**). Conversely, in Mexican Americans, a positive correlation was found between DM prevalence and flavonoid intake within the third quartile [1.04 (1.02, 1.07), *p* = 0.003] (**Table 3**). Moreover, the effect of flavonoid intake on the DM prevalence does not depend on the level of age, sex, BMI, daily energy intake, total time of PA, smoking habits, consumption of alcohol, hypertension history, and hyperlipidemia history (**Table 3**).

Age, sex, race, BMI, daily energy intake, total time of PA, smoking habits, consumption of alcohol, hypertension history, and hyperlipidemia history did not modify the impact of flavan-3-ols on the risk of DM (**Supplementary Table S3**). In individuals aged 50 years or older, a lower prevalence of DM was observed in those who consumed moderate to high levels of total flavan-3-ol (**Supplementary Table S3**). Specifically, consumption within the second quartile was associated with an OR of 0.95 (95% CI: 0.91, 0.99; *p* = 0.017), the third quartile with an OR of 0.94 (95% CI: 0.90, 0.98; *p* = 0.004), and the fourth quartile with an OR of 0.96 (95% CI: 0.92, 1.00; *p* = 0.035) (**Supplementary Table S3**). Males who consumed moderate to high amounts of flavan-3-ols demonstrated a lower occurrence of DM compared to those in the first quartile (**Supplementary Table S3**). Consumption within the third and fourth quartiles was associated with an OR of 0.97 (95% CI: 0.95, 1.00; *p* = 0.034) and 0.97 (95% CI: 0.94, 1.00; *p* = 0.025), respectively (**Supplementary Table S3**). Individuals with a BMI of 25.3 or greater exhibited a lower prevalence rate of DM when consuming moderate to high levels of total flavan-3-ols [within the third quartile 0.95 (0.92, 0.98), *p* = 0.001), and the fourth quartile [0.97 (0.94, 1.00), *p* = 0.046] (**Supplementary Table S3**).

Notably, the effect of subtotal catechin intake on the prevalence rate of DM was moderated by BMI (*p* for interaction = 0.047, **Supplementary Table S4**). Specifically, in individuals with a BMI >=25.3, the prevalence of DM was found to be lower in the third quartile of subtotal catechin intake [0.95 (0.93, 0.98), *p* = 0.001, **Supplementary Table S4**] and the fourth quartile of subtotal catechin intake [0.97 (0.94, 0.99), *p* = 0.018, **Supplementary Table S4**], compared to that in the first quartile. The prevalence rate of DM was lower in males who consumed subtotal catechin in the third quartile (OR = 0.97; 95% CI: 0.95, 1.00; *p* = 0.034, **Supplementary Table S4**) and in the fourth quartile (OR = 0.97; 95% CI: 0.94, 0.99; *p* = 0.014, **Supplementary Table S4**), compared to those in the first quartile. Intake within the second, third, and fourth quartiles of subtotal catechin consumption in the population age 50 or above were associated with lower occurrence rates of DM [0.96 (0.92, 1.00), *p* = 0.036; 0.94 (0.90, 0.99), *p* = 0.012; 0.96 (0.92, 1.00), *p* = 0.046, respectively], compared to those in the first quartile (**Supplementary Table S4**).

**Figure 3:**
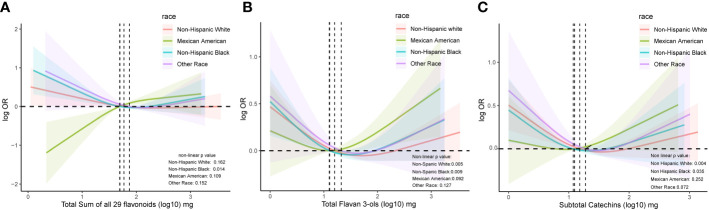
The association of flavonoid intake with prevalence of DM based on race by restricted cubic splines. Y axis stands for the log10 transformed Odds ratio of DM, and X axis stands for the log10 transformed intake of total flavonoids **(A)**, total flavan 3-ols **(B)**, and subtotal catechins **(C)**. Models by restricted cubic splines were adjusted for age, sex, race, BMI, daily energy intake, total time of PA, smoking status, alcohol consumption, total score of HEI, hypertension history and hyperlipidemia history.

Given the interaction between DM prevalence and race, RCS analysis was employed to examine the correlation between flavonoid intake and DM occurrence across different races. As shown in **Figure 3**, a statistically significant nonlinear correlation was noted between flavonoid consumption and DM prevalence in non-Hispanic Blacks (*p* for non-linearity = 0.014). Although the non-linear correlation was not found to be significant in the resting ethnic groups, a rising trend in DM risk with increasing total flavonoid intake was noted in Mexican Americans, which differed from the trends observed in other races. (**Figure 3**). Apart from Mexican Americans and other racial groups, significant U-shaped non-linear correlations were identified between flavan-3-ol consumption and DM prevalence in non-Hispanic Whites and non-Hispanic Blacks (*p* for non-linearity = 0.005 and 0.009, respectively, **Figure 3**). Similarly, significant U-shaped non-linear associations were observed between subtotal catechin intake and DM prevalence in Non-Hispanic Whites and Non-Hispanic Blacks (*p* for non-linearity = 0.004 and 0.035, respectively, **Figure 3**).

### 3.4 Sensitivity analysis

To further evaluate and improve the quality, credibility, and reproducibility of our results, the sensitivity analysis was performed, after excluding 732 (3.26%) of participants with IFG and 460 (2.05%) participants with IGT. Based on **Supplementary Table S5**, the prevalence of DM demonstrated an inverse correlation with the intake of total flavonoids in the second quartile [0.78 (0.63, 0.97), *p* = 0.025], third quartile [0.76 (0.60, 0.97), *p* = 0.03], and fourth quartile [0.80 (0.65, 0.98), *p* = 0.029] after complete adjustment, in comparison to the first quartile (**Supplementary Table S5**). However, there was no significant p-value for trend detected (*p* = 0.104) (**Supplementary Table S5**). Furthermore, a negative association between the prevalence of DM and the intake of total flavan 3-ols was found in the third quartile [0.65 (0.50, 0.84), *p* = 0.002], relative to that in the first quartile (**Supplementary Table S5**). Similarly, the prevalence of DM was negatively correlated with the consumption of subtotal catechins in the third quartile [0.70 (0.55, 0.89), *p* = 0.005], compared to that in the first quartile, as shown in **Supplementary Table S5**. The sensitivity analysis results showed that the associations between flavonoid intake and DM prevalence were minimally influenced by changes in inputs, which was consistent with **Table 2**. This suggests that our findings were robust and reliable.

## 4 Discussion

In 2021, the International Diabetes Federation (IDF) estimated that approximately 536.6 million adults between the ages of 20 and 79 have been diagnosed with DM worldwide, resulting in a global prevalence rate of 10.5% (26). By 2045, the prevalence of DM in adults is projected to reach 12.2% (26), which may be exacerbated post-COVID-19 pandemic (27). Diabetes-related mortality has been increasing globally, particularly in low- and middle-income countries (28). The diabetes-specific complications, especially the chronic complications of DM, impair the life quality and surge the demand for healthcare services and expenditures. The chronic complications of DM are divided into macroangiopathy, comprising stroke, coronary heart disease, and peripheral vascular disease, and microangiopathy; including end-stage renal disease, retinopathy, and neuropathy (29), which lead to the high prevalence of disability and mortality and impose a heavy burden on individuals and society.

However, prevention, early diagnosis, lifestyle modification, and disease course management exert a protective effect against the onset and progression of DM. Diets high in plants, containing micronutrients, fiber, and flavonoids, are routinely recommended in DM management (30). In line with our results showing a negative association between flavonoid intake and the risk of DM, several epidemiological investigations have documented an inverse correlation between dietary flavonoid consumption and the risk of DM. The findings from Eastern Europe indicated that flavonoid intake was negatively associated with fasting plasma glucose levels [0.71 (0.54–0.94)] (31) and T2D prevalence [0.44 (0.03–0.63)] (17). The US-based study demonstrated an inverse correlation between flavonoid and flavan-3-ol consumption and the occurrence of T2D in a 24-year follow-up of the NHS cohort (16). The consumption of total flavonoids was linked to a decreased prevalence of DM [0.67; (0.48, 0.93), *p* for trend = 0.02] during a mean follow-up period of 5.51 years in the PREDIMED (Prevención con Dieta Mediterránea) trial (18). Similarly, in the Europe population, total flavonoid intake was inversely related with T2D prevalence [0.90 (0.77–1.04), *p* for trend = 0.040, for the highest vs. the lowest quintile] (32). Furthermore, an increased dietary flavonoid intake was found to be associated with a reduced risk of diabetic nephropathy (33), revealing the potential protective role of flavonoids against diabetic complications.

However, there are many conflicting findings on the association between the prevalence of DM and flavonoid intake. The results from the US showed no association between the prevalence of DM and flavonoid intake in Song’s study (15), Nettleton’s study in postmenopausal women (14), and in the NHS II and HPFs cohorts (16). Apart from the differences in flavonoid intake among countries and regions, we suggest that the effects of flavonoids on glucose metabolism are significantly influenced by race. Through interaction analysis, we found an interaction between DM prevalence and total flavonoid intake (*p* for interaction = 0.006), implying different effects of flavonoids on DM prevalence in different ethnic subgroups. Further, the RCS analysis clearly showed that flavonoid intake differed in the risk of DM in different ethnic subgroups. Especially in the Mexican Americans, certain flavonoid intake levels were positively associated with the risk of DM.

The influence of ethnicity may be one of the reasons for the negative observations in the epidemiological studies from the US. Our study revealed a distinct association between total flavonoid intake and DM prevalence in Mexican Americans, which differed from that observed in the other racial groups. Specifically, among the Mexican American population, a notable positive association was observed between the prevalence of DM and total flavonoid intake within the third quartile. Further, the mechanisms by which race might impact the association between flavonoid intake and DM prevalence may involve genetic predisposition, dietary practices, and lifestyle behaviors. When considering genetic predisposition, it is worth noting that SLC16A11 has been recognized as a risk factor for T2D in individuals of Mexican American descent (34). This variant occurs at a frequency of approximately 50% in Native American populations and around 10% in populations of East Asian ancestry, whereas it is uncommon among individuals of European and African descent (34). Furthermore, genetic variations related to ethnicity, such as differences in the intestinal microbiota and phase I and II metabolism, may also impact the bioavailability of flavonoids (35). Furthermore, the unique dietary and lifestyle habits observed in Mexican Americans within our study included the lowest consumption of flavonoid levels and the highest proportion of heavy alcohol consumption. Thus, additional research is necessary to explore the potential influence of dietary flavonoids on diabetes mellitus risk in diverse ethnic populations, as well as whether genetic variants such as SLC16A11 could influence the absorption and metabolism of flavonoids and their association with DM prevalence specifically among Mexican Americans.

Certain dietary flavonoids have been demonstrated to have a negative correlation with the prevalence of DM, according to epidemiological research (36–38). The consumption of flavan-3-ols was marginally associated with a reduced prevalence of T2D [0.89 (0.80, 1.00), *p* for trend = 0.06) in the Framingham Offspring cohort (13). The consumption of each individual flavan-3-ol monomer was significantly linked to T2D risk in European populations (32, 39). A clinical trial demonstrated a negative correlation between fasting glucose concentration and consumption of flavonoids as well as flavan-3-ols (40). A meta-analysis demonstrated that an increase of 68 mg/day in flavan-3-ol consumption was linked to a 6% decrease in the risk of T2D [(0.92, 0.96), *p* for trend <0.001] (38). In line with previous studies, our findings indicate that flavan-3-ols, particularly catechins, are the predominant flavonoids related to a decreased risk of DM. Flavan-3-ols are enriched in tea, cocoa, fruits, and nuts (41, 42). Possible mechanisms underlying the ability of flavan-3-ols to lower the risk of DM involve scavenging of free radicals, inhibition of inflammation, and antioxidant responses involved in glucose metabolism (30).

Flavan-3-ols have subclasses that include subtotal catechins (epicatechin, epicatechin 3-gallate, epigallocatechin, epigallocatechin 3-gallate, catechin, and gallocatechin) and theaflavins. Relatively few studies on the association between catechins and DM. In the PREDIMED trial, the intake of catechins in the middle tertile, not in the highest tertile, was inversely associated with DM risk (18), which could imply a non-linear relationship or U-shaped correlation between catechin consumption and the risk of DM. Consistent with previous findings, we identified a negative correlation between the consumption of catechins within the intermediate levels and the risk of DM. Catechins are reported to reduce insulin resistance, mitigate endoplasmic reticulum stress and oxidative stress, alleviate inflammatory response, improve mitochondrial function, and improve intestinal and microbial function (43).

Our study may provide preliminary findings for the prevention of DM. Secondary prevention is a method of identifying the illness by means of targeted screening measures and routine medical monitoring (44). The American Diabetes Association (ADA) highlights the significance of screening for diabetes, as diabetes often lacks recognizable symptoms. In the absence of efficient screening initiatives, individuals with high risk are less likely to undertake the essential intervention to prevent or delay the progression to T2D (45). Tertiary prevention refers to the measures aimed at individuals who have already experienced disease or injury, with the goal of mitigating the consequences of the condition, slowing down disease progression, and enhancing quality of life (44). The majority of medications designed to delay the onset of diabetes are associated with considerable expenses, while a substantial portion remains uncovered by insurance providers due to the lack of an approved indication by the Food and Drug Administration (FDA) (45). Lifestyle interventions demonstrate cost-effectiveness in the prevention of diabetes. The lifestyle intervention targeting the reduction of caloric intake from fat and an increase in dietary fiber intake demonstrated a notable decrease in the occurrence of diabetes (33, 46–48). Our findings suggested that increasing the intake of flavan-3-ol and catechin within a certain range may reduce the prevalence of diabetes. Along with other findings, plant-based diets may play an important role in diabetes prevention. Therefore, we propose potential public health policy measures that could be established based on our results. Enhancing public knowledge and awareness of plant-based diets through educational and promotional activities, promoting dietary guidelines and nutritional recommendations. Providing plant-based diet choices in schools and workplaces. Encouraging the consumption of plant-based diets and increasing the purchase and consumption of plant-based foods through price controls and taxation policies. Establishing a sustainable supply chain for plant-based diets, promoting agricultural sustainability and organic farming, and ensuring easy access to and affordability of plant-based food options. Implementing policies or regulations that require food manufacturers to label nutritional information such as the contents of flavonoids on food products.

In our study, we detected a curvilinear association between the prevalence of diabetes and total flavonoid intake. More precisely, the likelihood of developing DM was observed to increase with the intake of flavan 3-ols and subtotal catechins at specific levels in our study. This phenomenon could be attributed to certain flavonoids that exhibit pro-oxidant effects under specific conditions, including the presence of transition metals such as iron or copper (49). This process could give rise to the production of reactive oxygen species (ROS), which can promote oxidative stress and damage various cellular constituents, such as proteins, lipids, and DNA (50). For instance, epigallocatechin gallate, which is classified as a catechin subclass, was reported to provoke H_2_O_2_ production and consequent oxidative damage to cellular DNA when exposed to transition metal ions (51). Furthermore, flavonoids that feature a phenol B ring, such as apigenin and naringenin, undergo peroxidase/H_2_O_2_-driven oxidation to generate phenoxyl radicals, which promote co-oxidation of GSH or NADH, ultimately resulting in the production of ROS (52, 53). Although flavonoids have beneficial effects, it is crucial to carefully evaluate the impact of the potential pro-oxidant activity of flavonoids on health outcomes.

There are several limitations of our study. The observational study does not allow causal inferences to be made, therefore, randomized controlled clinical trials should be conducted. Importantly, future prospective studies with larger sample sizes and diverse ethnic cohorts are warranted

## 5 Conclusion

In summary, our study demonstrated that specific consumptions of flavonoids were inversely correlated with the risk of DM, with ethnicity exerting a modifying effect on this relationship. We observed a negative correlation between the risk of diabetes and moderate intake of flavan-3-ols and catechins only. Our results may provide valuable information for customized nutritional interventions in the context of DM management.

## Data availability statement

The original contributions presented in the study are included in the article/**Supplementary Material.** Further inquiries can be directed to the corresponding author.

## Ethics statement

The studies involving humans were approved by The NHANES study was conducted in accordance with the Declaration of Helsinki, and approved by the Institutional Review Board (or Ethics Committee) of the institutional review board of the National Center for Health Statistics, CDC (protocol #2005-06, #2011-17, #2018-01). The studies were conducted in accordance with the local legislation and institutional requirements. The participants provided their written informed consent to participate in this study.

## Author contributions

Conceptualization, YJZ and PX. Methodology, YJZ and PX. Validation, YJZ and SQ. Formal analysis, YJZ and PX. Writing— original draft preparation, YJZ and SQ. Writing—review and editing YJZ. visualization, YJZ, YZ, and SQ. Supervision, YJZ and KG. Project administration, YJZ and PX. Funding acquisition, YJZ and KG. All authors contributed to the article and approved the submitted version.
