# Supplementary material for: The associations between dietary flavonoid intake and the prevalence of diabetes mellitus: Data from the National Health and Nutrition Examination Survey 2007-2010 and 2017-2018
Source: Front Endocrinol (Lausanne). 2023 Aug 19;14:1250410. doi: 10.3389/fendo.2023.1250410 (PMC10474301; doi:10.3389/fendo.2023.1250410)
Supplement: Supplementary file 1 [file DataSheet_1.docx]

Supplementary Material

The associations between dietary flavonoid intake and the prevalence of diabetes mellitus in the National Health and Nutrition Examination Survey 2007–2010 and 2017–2018

Yanjun Zhou^1#^, Peng Xu^2#^, Shaolei Qin ^3^, Yan Zhu ^1^, Ke Gu ^1,^*

1 Department of Radiotherapy and Oncology, The Affiliated Hospital of Jiangnan University, Wuxi, Jiangsu, China

2 Population Health Sciences, German Center for Neurodegenerative Diseases (DZNE), Bonn, Gemany

3 Jiangnan University, Wuxi, Jiangsu, China

# These authors contributed to the work equally and should be regarded as co-first authors.

* Correspondence:
Corresponding Author
yourprofGUKE@126.com

# Supplementary Figures


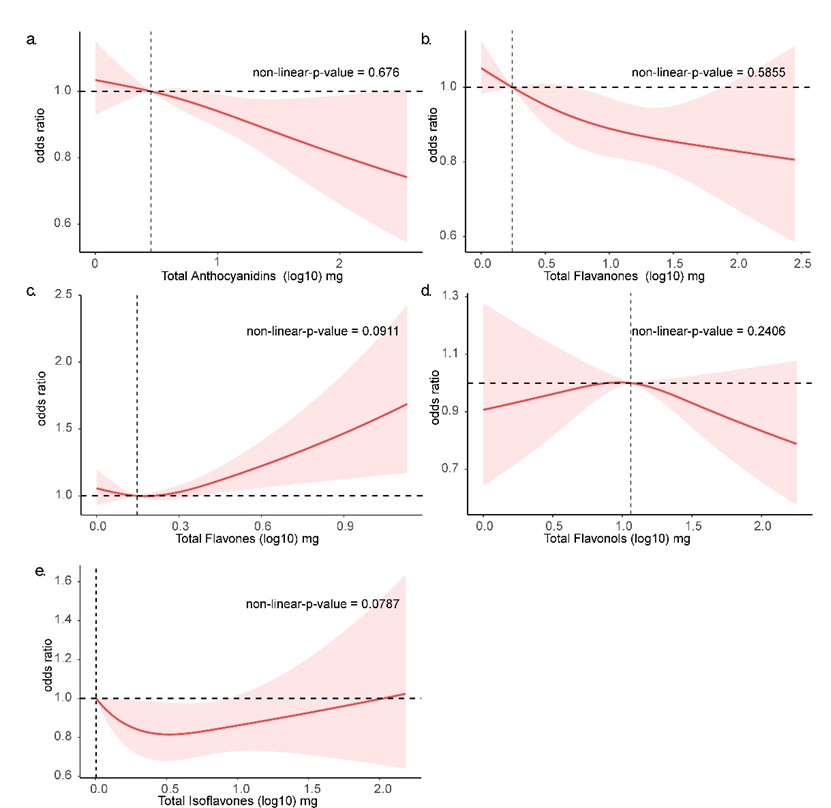


**Supplementary Figure S1.** The association of flavonoid intake with incidence of DM by restricted cubic splines. Y axis stands for the Odds ratio of DM, and X axis stands for the log10 transformed intake of total anthocyanidines (a), total flavanones (b), total flavones (c), total flavonols (d), and total isoflavones (e). Models by restricted cubic splines were adjusted for age, sex, race, BMI, daily energy intake, total time of PA, smoking status, alcohol consumption, total score of HEI, hypertension history and hyperlipidaemia history.

# Supplementary Tables

**Supplementary Table S1.** The demographic features of the study cohort grouped by racial category.

| Variables | Mexican American | Non-Hispanic White | Non-Hispanic Black | Other Race | p value |
| --- | --- | --- | --- | --- | --- |
|  | N = 4291 | N = 11511 | N = 4771 | N = 1908 |  |
| Age (Years) | 28.27(0.56) | 39.84(0.36) | 34.10(0.45) | 33.42(0.80) | < 0.0001 |
| Sex |  |  |  |  | 0.04 |
| Female | 2234(50.68%) | 5819(51.75%) | 2472(54.48%) | 955(51.06%) |  |
| Male | 2057(49.32%) | 5692(48.25%) | 2299(45.52%) | 953(48.94%) |  |
| Education |  |  |  |  | < 0.0001 |
| <9 years | 916(17.08%) | 793(3.43%) | 263(3.93%) | 86(3.62%) |  |
| 9-12 years | 1790(43.39%) | 4507(34.72%) | 2126(45.30%) | 501(27.50%) |  |
| >12 years | 1585(39.53%) | 6211(61.86%) | 2382(50.77%) | 1321(68.88%) |  |
| Marital status |  |  |  |  | < 0.0001 |
| Without partner | 2261(48.70%) | 5543(43.38%) | 3014(63.53%) | 922(45.87%) |  |
| With partner | 2030(51.30%) | 5968(56.62%) | 1757(36.47%) | 986(54.13%) |  |
| Smoking status |  |  |  |  | < 0.0001 |
| Former | 471(19.29%) | 2275(26.92%) | 579(15.57%) | 192(19.48%) |  |
| Never | 1429(65.30%) | 3915(54.28%) | 1625(58.92%) | 785(65.55%) |  |
| Now | 323(15.41%) | 1583(18.80%) | 713(25.51%) | 168(14.97%) |  |
| Alcohol consumption |  |  |  |  | < 0.0001 |
| Former | 294(11.50%) | 1126(11.42%) | 364(11.12%) | 53(7.72%) |  |
| Heavy | 584(34.48%) | 1374(21.19%) | 417(19.53%) | 137(16.97%) |  |
| Mild | 477(23.15%) | 2571(39.79%) | 913(36.37%) | 427(43.80%) |  |
| Moderate | 298(16.34%) | 1114(18.69%) | 464(19.35%) | 116(11.40%) |  |
| Never | 325(14.52%) | 810(8.90%) | 355(13.63%) | 225(20.11%) |  |
| BMI (kg/m^2^) | 26.68(0.21) | 26.86(0.13) | 28.10(0.15) | 25.10(0.31) | < 0.0001 |
| Total score of HEI 2015 | 52.41(0.39) | 52.47(0.39) | 50.11(0.39) | 54.07(0.45) | < 0.0001 |
| DII | 1.67(0.07) | 1.62(0.05) | 2.08(0.04) | 1.55(0.08) | < 0.0001 |
| Total time of PA (mins/week) | 1367.63(37.80) | 1240.56(31.31) | 1373.24(33.62) | 1133.85(62.60) | < 0.001 |
| Total MET of PA (/week) | 5869.83(239.90) | 4915.90(154.03) | 5668.97(194.56) | 4419.91(328.78) | < 0.0001 |
| Daily energy intake (kcal) | 3884.98(40.68) | 4087.16(26.25) | 3915.75(36.08) | 3914.64(60.54) | < 0.0001 |
| PIR | 1.87(0.05) | 3.16(0.05) | 2.16(0.05) | 2.86(0.11) | < 0.0001 |
| Hyperlipidemia |  |  |  |  | < 0.0001 |
| No | 1401(38.51%) | 3599(36.61%) | 2051(49.53%) | 743(43.02%) |  |
| Yes | 2097(61.49%) | 6468(63.39%) | 2108(50.47%) | 901(56.98%) |  |
| CVD |  |  |  |  | < 0.001 |
| No | 2025(95.05%) | 6618(90.67%) | 2529(90.73%) | 1024(92.88%) |  |
| Yes | 156(4.95%) | 1069(9.33%) | 352(9.27%) | 83(7.12%) |  |
| COPD and asthma |  |  |  |  | < 0.0001 |
| COPD and asthma | 19(0.58%) | 247(2.43%) | 63(1.60%) | 23(1.97%) |  |
| Asthma | 162(7.56%) | 919(11.34%) | 457(17.75%) | 143(13.65%) |  |
| COPD | 22(0.48%) | 362(3.66%) | 84(2.15%) | 16(1.34%) |  |
| No | 2037(91.38%) | 6273(82.57%) | 2330(78.50%) | 939(83.04%) |  |
| Stroke |  |  |  |  | 0.002 |
| No | 2125(98.28%) | 7314(96.74%) | 2703(95.06%) | 1068(97.05%) |  |
| Yes | 55(1.72%) | 359(3.26%) | 175(4.94%) | 37(2.95%) |  |
| Cancer |  |  |  |  | < 0.0001 |
| No | 2079(96.66%) | 6588(88.24%) | 2680(94.76%) | 1035(94.60%) |  |
| Yes | 100(3.34%) | 1091(11.76%) | 200(5.24%) | 71(5.40%) |  |
| Hypertension |  |  |  |  | < 0.0001 |
| No | 3548(84.33%) | 8078(70.93%) | 3180(67.87%) | 1490(77.08%) |  |
| Yes | 743(15.67%) | 3433(29.07%) | 1591(32.13%) | 418(22.92%) |  |
| DM |  |  |  |  | 0.03 |
| No | 3810(90.58%) | 10125(89.68%) | 4081(87.47%) | 1701(89.82%) |  |
| Yes | 481(9.42%) | 1386(10.32%) | 690(12.53%) | 207(10.18%) |  |
| Dietary intake of flavonoids (mg/day) | | |  |  |  |
| Daidzein | 0.58(0.17) | 0.70(0.06) | 0.49(0.06) | 0.97(0.14) | 0.01 |
| Genistein | 0.86(0.24) | 1.00(0.08) | 0.65(0.08) | 1.27(0.17) | 0.004 |
| Glycitein | 0.12(0.04) | 0.15(0.01) | 0.09(0.01) | 0.17(0.02) | 0.01 |
| Cyanidin | 1.84(0.17) | 2.73(0.20) | 1.33(0.11) | 2.43(0.27) | < 0.0001 |
| Petunidin | 0.54(0.07) | 1.24(0.12) | 0.48(0.05) | 0.96(0.13) | < 0.0001 |
| Delphinidin | 0.74(0.09) | 1.68(0.15) | 0.65(0.06) | 2.12(0.48) | < 0.0001 |
| Malvidin | 2.27(0.20) | 5.02(0.37) | 3.23(0.22) | 3.88(0.42) | < 0.0001 |
| Pelargonidin | 1.14(0.09) | 1.91(0.18) | 0.89(0.10) | 1.56(0.16) | < 0.0001 |
| Peonidin | 1.06(0.25) | 1.99(0.17) | 1.03(0.14) | 2.10(0.66) | 0.002 |
| Catechin | 5.40(0.17) | 7.52(0.20) | 5.13(0.12) | 7.60(0.36) | < 0.0001 |
| Epigallocatechin | 6.59(0.65) | 14.92(0.87) | 8.88(0.50) | 17.72(1.73) | < 0.0001 |
| Epicatechin | 7.73(0.26) | 9.96(0.27) | 7.97(0.22) | 10.90(0.65) | < 0.0001 |
| Epicatechin 3 gallate | 4.06(0.42) | 9.64(0.56) | 5.71(0.33) | 11.40(1.08) | < 0.0001 |
| Epigallocatechin 3 gallate | 11.78(1.23) | 25.47(1.74) | 14.93(0.87) | 31.69(3.87) | < 0.0001 |
| Theaflavin | 0.50(0.06) | 1.45(0.08) | 0.87(0.06) | 1.53(0.12) | < 0.0001 |
| Thearubigins | 30.64(3.53) | 83.51(4.62) | 50.52(3.06) | 83.08(6.29) | < 0.0001 |
| Eriodictyol | 0.22(0.02) | 0.14(0.01) | 0.22(0.01) | 0.19(0.03) | < 0.0001 |
| Hesperetin | 11.87(0.54) | 8.15(0.26) | 10.25(0.46) | 9.72(0.87) | < 0.0001 |
| Naringenin | 4.22(0.28) | 2.95(0.15) | 3.42(0.15) | 3.64(0.36) | < 0.001 |
| Apigenin | 0.13(0.02) | 0.22(0.03) | 0.11(0.01) | 0.18(0.02) | < 0.0001 |
| Luteolin | 0.64(0.03) | 0.63(0.02) | 0.40(0.02) | 0.78(0.04) | < 0.0001 |
| Isorhamnetin | 0.72(0.03) | 0.73(0.02) | 0.55(0.03) | 0.93(0.06) | < 0.0001 |
| Kaempferol | 2.56(0.10) | 4.10(0.11) | 3.20(0.11) | 4.43(0.19) | < 0.0001 |
| Myricetin | 0.83(0.05) | 1.40(0.05) | 0.92(0.04) | 1.36(0.08) | < 0.0001 |
| Quercetin | 8.35(0.24) | 10.43(0.24) | 7.83(0.13) | 11.24(0.39) | < 0.0001 |
| Theaflavin 3 3 digallate | 0.54(0.06) | 1.60(0.09) | 0.96(0.06) | 1.68(0.14) | < 0.0001 |
| Theaflavin 3q gallate | 0.46(0.05) | 1.36(0.08) | 0.82(0.05) | 1.44(0.12) | < 0.0001 |
| Theaflavin 3 gallate | 0.39(0.05) | 1.15(0.07) | 0.69(0.04) | 1.20(0.10) | < 0.0001 |
| Gallocatechin | 0.61(0.06) | 1.54(0.08) | 0.92(0.06) | 1.70(0.13) | < 0.0001 |
| Subtotal Catechins | 36.19(2.63) | 69.04(3.59) | 43.54(1.99) | 81.02(7.60) | < 0.0001 |
| Total Isoflavones | 1.56(0.45) | 1.85(0.15) | 1.24(0.16) | 2.42(0.33) | 0.01 |
| Total Anthocyanidins | 7.59(0.68) | 14.57(0.85) | 7.61(0.46) | 13.06(1.34) | < 0.0001 |
| Total Flavan 3-ols | 68.71(6.15) | 158.12(7.74) | 97.40(5.02) | 169.93(11.80) | < 0.0001 |
| Total Flavanones | 16.31(0.73) | 11.23(0.39) | 13.89(0.55) | 13.55(1.19) | < 0.0001 |
| Total Flavones | 0.77(0.03) | 0.85(0.04) | 0.52(0.02) | 0.96(0.04) | < 0.0001 |
| Total Flavonols | 12.46(0.36) | 16.67(0.40) | 12.50(0.27) | 17.96(0.61) | < 0.0001 |
| Total Sum of all 29 flavonoids | 107.40(6.38) | 203.28(8.22) | 133.17(5.11) | 217.88(12.36) | < 0.0001 |

PIR: poverty income ratio; BMI: body mass index; DII: dietary inflammatory index; HEI: healthy eating index. Continuous normal variables were presented as weighted mean ± standard deviation, One-way ANOVA was employed to compare difference between groups. Categorical variables were presented as frequencies and percentages and compared using Chi-Squared test.

**Supplementary Table S2.** The characteristics of study population stratified by total flavonoid intake quartiles.

| Variables | 1Q | 2Q | 3Q | 4Q | p-value |
| --- | --- | --- | --- | --- | --- |
|  | N = 5624 | N = 5618 | N = 5619 | N = 5620 |  |
| Age (years) | 32.83(0.55) | 33.23(0.55) | 37.26(0.48) | 44.25(0.45) | < 0.0001 |
| Sex |  |  |  |  | 0.3 |
| Female | 2850(51.45%) | 2869(50.94%) | 2813(51.29%) | 2948(53.49%) |  |
| Male | 2774(48.55%) | 2749(49.06%) | 2806(48.71%) | 2672(46.51%) |  |
| Ethnicity |  |  |  |  | < 0.0001 |
| Non-Hispanic White | 2820(68.45%) | 2738(67.38%) | 2765(68.31%) | 3188(74.49%) |  |
| Non-Hispanic Black | 1314(14.31%) | 1219(12.80%) | 1127(12.09%) | 1111(9.82%) |  |
| Mexican American | 1101(11.30%) | 1232(12.68%) | 1262(12.66%) | 696(6.09%) |  |
| Other Race | 389(5.94%) | 429(7.15%) | 465(6.94%) | 625(9.60%) |  |
| Education |  |  |  |  | < 0.0001 |
| <9 years | 583(6.29%) | 574(5.47%) | 544(5.13%) | 357(3.30%) |  |
| 9-12 years | 2783(48.09%) | 2244(36.20%) | 1954(31.80%) | 1943(31.31%) |  |
| >12 years | 2258(45.61%) | 2800(58.32%) | 3121(63.08%) | 3320(65.38%) |  |
| Marital status |  |  |  |  | < 0.0001 |
| Without partner | 3301(53.11%) | 3146(51.29%) | 2886(45.68%) | 2407(38.81%) |  |
| With partner | 2323(46.89%) | 2472(48.71%) | 2733(54.32%) | 3213(61.19%) |  |
| Smoking status |  |  |  |  | < 0.0001 |
| Never | 1421(46.29%) | 1657(56.86%) | 2004(60.56%) | 2672(59.75%) |  |
| Past | 709(23.68%) | 765(24.04%) | 896(25.33%) | 1147(24.47%) |  |
| Current | 895(30.03%) | 614(19.10%) | 506(14.11%) | 772(15.78%) |  |
| Alcohol consumption |  |  |  |  | < 0.0001 |
| Never | 363(10.95%) | 333(8.78%) | 440(11.92%) | 579(10.81%) |  |
| Former | 510(14.96%) | 387(11.05%) | 365(8.78%) | 575(10.58%) |  |
| Mild | 717(28.64%) | 895(36.39%) | 1148(40.54%) | 1628(43.14%) |  |
| Moderate | 429(19.10%) | 432(17.09%) | 483(18.56%) | 648(17.70%) |  |
| Heavy | 611(26.34%) | 637(26.69%) | 589(20.19%) | 675(17.77%) |  |
| Daily energy intake (kcal/day) | 3511.58(30.23) | 3984.08(34.56) | 4230.20(31.40) | 4299.92(38.85) | < 0.0001 |
| PIR | 2.50(0.05) | 2.78(0.05) | 2.94(0.05) | 3.21(0.05) | < 0.0001 |
| BMI (kg/m^2^) | 27.10(0.18) | 25.87(0.17) | 26.30(0.19) | 27.86(0.16) | < 0.0001 |
| Total score of HEI (2015) | 43.91(0.27) | 52.22(0.28) | 56.93(0.30) | 54.94(0.46) | < 0.0001 |
| DII | 2.63(0.04) | 1.86(0.04) | 1.17(0.04) | 1.23(0.06) | < 0.0001 |
| Total time of PA (mins/week) | 1416.25(49.45) | 1237.63(35.07) | 1168.26(29.19) | 1238.36(41.75) | < 0.0001 |
| Total MET of PA (/week) | 5828.21(245.93) | 5037.67(206.66) | 4617.09(141.37) | 4887.95(179.39) | < 0.001 |
| Hyperlipidemia |  |  |  |  | 0.003 |
| No | 1921(40.64%) | 2030(41.66%) | 1937(38.34%) | 1906(36.00%) |  |
| Yes | 2615(59.36%) | 2588(58.34%) | 2844(61.66%) | 3527(64.00%) |  |
| CVD |  |  |  |  | 0.35 |
| No | 2546(90.29%) | 579(10.81%) | 3005(92.02%) | 4018(91.37%) |  |
| Yes | 399(9.71%) | 368(9.11%) | 362(7.98%) | 531(8.63%) |  |
| COPD and asthma |  |  |  |  | 0.11 |
| COPD and asthma | 92(2.54%) | 81(2.05%) | 80(1.88%) | 99(2.14%) |  |
| Asthma | 412(14.08%) | 365(11.84%) | 387(11.60%) | 517(10.97%) |  |
| COPD | 133(3.54%) | 101(2.85%) | 96(2.90%) | 154(2.96%) |  |
| No | 2370(79.84%) | 2530(83.26%) | 2868(83.62%) | 3811(83.92%) |  |
| Stroke |  |  |  |  | 0.16 |
| No | 2784(96.02%) | 2843(96.26%) | 3228(97.44%) | 4355(96.86%) |  |
| Yes | 154(3.98%) | 150(3.74%) | 132(2.56%) | 190(3.14%) |  |
| Cancer |  |  |  |  | 0.01 |
| No | 2663(91.37%) | 2702(91.93%) | 2989(88.30%) | 4028(89.69%) |  |
| Yes | 278(8.63%) | 290(8.07%) | 375(11.70%) | 519(10.31%) |  |
| Hypertension |  |  |  |  | < 0.0001 |
| No | 4277(74.67%) | 4274(75.09%) | 4118(73.36%) | 3627(68.00%) |  |
| Yes | 1347(25.33%) | 1344(24.91%) | 1501(26.64%) | 1993(32.00%) |  |

PIR: poverty income ratio; BMI: body mass index; DII: dietary inflammatory index; HEI: healthy eating index. Continuous normal variables were presented as weighted mean ± standard deviation, One-way ANOVA was employed to compare difference between groups. Categorical variables were presented as frequencies and percentages and compared using Chi-Squared test.

**Supplementary Table S3**. Stratified association between DM incidence and total flavan 3-ol intake in the NHANSE (2007-2010 and 2017-2018).

| Total flavan 3-ol intake (mg/day) | Q1 (≤4.605) | Q2 (4.605-13.245) |  | Q3 (13.245-59.525) |  | Q4 (>59.525) |  | p for interaction |
| --- | --- | --- | --- | --- | --- | --- | --- | --- |
|  | N = 5624 | N = 5615 |  | N = 5622 |  | N = 5620 |  |  |
|  |  | OR (95%CI) | p value | OR (95%CI) | p value | OR (95%CI) | p value |  |
| Age (Years) |  |  |  |  |  |  |  | 0.415 |
| <50 | Ref | 1.00 (0.98, 1.01) | 0.483 | 0.99 (0.97, 1.00) | 0.061 | 1.00 (0.98, 1.01) | 0.508 |  |
| >=50 | Ref | 0.95 (0.91, 0.99) | 0.017 | 0.94 (0.90, 0.98) | 0.004 | 0.96 (0.92, 1.00) | 0.035 |  |
| Sex |  |  |  |  |  |  |  | 0.600 |
| Female | Ref | 1.00 (0.98, 1.03) | 0.877 | 0.98 (0.96, 1.01) | 0.219 | 1.00 (0.97, 1.02) | 0.682 |  |
| Male | Ref | 0.98 (0.95, 1.00) | 0.079 | 0.97 (0.95, 1.00) | 0.034 | 0.97 (0.94, 1.00) | 0.025 |  |
| Race |  |  |  |  |  |  |  | 0.135 |
| Non-Hispanic White | Ref | 0.99 (0.96, 1.02) | 0.408 | 0.98 (0.96, 1.01) | 0.161 | 0.98 (0.95, 1.01) | 0.134 |  |
| Non-Hispanic Black | Ref | 0.98 (0.95, 1.02) | 0.353 | 0.97 (0.93, 1.00) | 0.043 | 0.98 (0.95, 1.01) | 0.19 |  |
| Mexican American | Ref | 0.99 (0.96, 1.03) | 0.693 | 0.99 (0.96, 1.02) | 0.498 | 1.03 (0.98, 1.08) | 0.246 |  |
| Other Race | Ref | 1.02 (0.96, 1.07) | 0.531 | 0.96 (0.92, 1.01) | 0.095 | 0.98 (0.92, 1.04) | 0.533 |  |
| BMI (kg/m2) |  |  |  |  |  |  |  | 0.090 |
| <25.3 | Ref | 1.00 (0.98, 1.01) | 0.918 | 1.00 (0.99, 1.02) | 0.939 | 1.00 (0.99, 1.02) | 0.789 |  |
| >=25.3 | Ref | 0.97 (0.94, 1.00) | 0.05 | 0.95 (0.92, 0.98) | 0.001 | 0.97 (0.94, 1.00) | 0.046 |  |
| Daily energy intake (kcal) |  |  |  |  |  |  |  | 0.140 |
| <3584 | Ref | 0.98 (0.95, 1.01) | 0.16 | 0.97 (0.94, 1.00) | 0.029 | 0.98 (0.95, 1.01) | 0.195 |  |
| >=3584 | Ref | 1.00 (0.97, 1.02) | 0.694 | 0.98 (0.96, 1.00) | 0.091 | 0.98 (0.95, 1.01) | 0.167 |  |
| Total time of PA (mins/week) |  |  |  |  |  |  |  | 0.479 |
| <600 | Ref | 0.99 (0.95, 1.02) | 0.477 | 0.97 (0.93, 1.00) | 0.053 | 0.97 (0.94, 1.00) | 0.025 |  |
| >=600 | Ref | 0.99 (0.97, 1.01) | 0.462 | 0.99 (0.97, 1.01) | 0.442 | 1.00 (0.98, 1.03) | 0.925 |  |
| Smoking habits |  |  |  |  |  |  |  | 0.527 |
| Never | Ref | 0.99 (0.96, 1.01) | 0.324 | 0.98 (0.96, 1.00) | 0.064 | 0.98 (0.96, 1.01) | 0.157 |  |
| Former | Ref | 0.96 (0.93, 1.01) | 0.086 | 0.95 (0.92, 0.99) | 0.014 | 0.99 (0.95, 1.03) | 0.5 |  |
| Current | Ref | 1.01 (0.97, 1.06) | 0.47 | 1.00 (0.96, 1.04) | 0.95 | 0.98 (0.95, 1.02) | 0.353 |  |
| Alcohol Consumption | |  |  |  |  |  |  | 0.630 |
| Never | Ref | 0.96 (0.91, 1.02) | 0.169 | 0.98 (0.93, 1.04) | 0.578 | 0.99 (0.94, 1.05) | 0.694 |  |
| Former | Ref | 1.01 (0.96, 1.07) | 0.649 | 1.00 (0.95, 1.05) | 0.858 | 0.96 (0.91, 1.01) | 0.102 |  |
| Mild | Ref | 0.98 (0.93, 1.03) | 0.388 | 0.94 (0.90, 0.99) | 0.019 | 0.97 (0.92, 1.01) | 0.142 |  |
| Moderate | Ref | 0.99 (0.92, 1.06) | 0.776 | 0.99 (0.93, 1.06) | 0.808 | 1.04 (0.97, 1.12) | 0.294 |  |
| Heavy | Ref | 0.98 (0.93, 1.03) | 0.387 | 0.95 (0.91, 0.99) | 0.018 | 0.98 (0.94, 1.02) | 0.251 |  |
| Total score of HEI (2015 Edition) | |  |  |  |  |  |  | 0.445 |
| <51.44187 | Ref | 0.99 (0.96, 1.02) | 0.368 | 0.98 (0.95, 1.00) | 0.091 | 0.97 (0.94, 0.99) | 0.013 |  |
| >=51.44187 | Ref | 1.00 (0.98, 1.03) | 0.732 | 1.00 (0.97, 1.02) | 0.818 | 1.01 (0.99, 1.04) | 0.321 |  |
| Hypertension |  |  |  |  |  |  |  | 0.634 |
| No | Ref | 0.99 (0.98, 1.01) | 0.54 | 0.99 (0.98, 1.01) | 0.54 | 0.99 (0.98, 1.01) | 0.581 |  |
| Yes | Ref | 0.96 (0.90, 1.01) | 0.125 | 0.91 (0.87, 0.96) | 0.001 | 0.95 (0.91, 1.00) | 0.055 |  |
| Hyperlipidemia |  |  |  |  |  |  |  | 0.375 |
| No | Ref | 0.99 (0.98, 1.01) | 0.216 | 0.98 (0.97, 1.00) | 0.012 | 0.99 (0.97, 1.01) | 0.337 |  |
| Yes | Ref | 0.99 (0.95, 1.02) | 0.451 | 0.97 (0.94, 1.01) | 0.102 | 0.98 (0.95, 1.01) | 0.249 |  |

BMI: body mass index; HEI: healthy eating index; PA: physical activity.

**Supplementary Table S4**. Stratified association between DM incidence and subtotal catechin intake in the NHANSE (2007-2010 and 2017-2018).

|  | Q1 (≤4.505) | Q2 (4.505-12.745) |  | Q3 (12.745-37.725) |  | Q4 (>37.725) |  | p for interaction |
| --- | --- | --- | --- | --- | --- | --- | --- | --- |
|  | N = 5619 | N = 5622 |  | N = 5621 |  | N = 5619 |  |  |
|  |  | OR (95%CI) | p value | OR (95%CI) | p value | OR (95%CI) | p value |  |
| Age (Years) |  |  |  |  |  |  |  | 0.152 |
| <50 | Ref | 1.00 (0.98, 1.01) | 0.565 | 0.99 (0.97, 1.00) | 0.058 | 0.99 (0.98, 1.01) | 0.341 |  |
| >=50 | Ref | 0.96 (0.92, 1.00) | 0.036 | 0.94 (0.90, 0.99) | 0.012 | 0.96 (0.92, 1.00) | 0.046 |  |
| Gender |  |  |  |  |  |  |  | 0.791 |
| Female | Ref | 1.00 (0.98, 1.03) | 0.708 | 0.99 (0.97, 1.02) | 0.532 | 0.99 (0.97, 1.02) | 0.606 |  |
| Male | Ref | 0.98 (0.95, 1.01) | 0.106 | 0.97 (0.95, 1.00) | 0.034 | 0.97 (0.94, 0.99) | 0.014 |  |
| Race |  |  |  |  |  |  |  | 0.125 |
| Non-HispanicWhite | Ref | 0.99 (0.96, 1.02) | 0.571 | 0.99 (0.96, 1.01) | 0.216 | 0.98 (0.95, 1.00) | 0.087 |  |
| Non-Hispanic Black | Ref | 0.98 (0.95, 1.01) | 0.243 | 0.98 (0.95, 1.02) | 0.269 | 0.98 (0.95, 1.01) | 0.148 |  |
| Mexican American | Ref | 0.99 (0.95, 1.03) | 0.601 | 0.99 (0.96, 1.03) | 0.735 | 1.02 (0.98, 1.07) | 0.299 |  |
| Other Race | Ref | 1.02 (0.97, 1.08) | 0.364 | 0.96 (0.93, 1.00) | 0.076 | 0.98 (0.92, 1.04) | 0.518 |  |
| BMI (kg/m2) |  |  |  |  |  |  |  | 0.047 |
| <25.3 | Ref | 1.00 (0.98, 1.01) | 0.817 | 1.00 (0.99, 1.02) | 0.527 | 1.00 (0.99, 1.01) | 0.995 |  |
| >=25.3 | Ref | 0.98 (0.95, 1.00) | 0.101 | 0.95 (0.93, 0.98) | 0.001 | 0.97 (0.94, 0.99) | 0.018 |  |
| Daily energy intake (kcal) |  |  |  |  |  |  |  | 0.318 |
| <3584 | Ref | 0.99 (0.96, 1.02) | 0.379 | 0.98 (0.95, 1.01) | 0.144 | 0.98 (0.95, 1.00) | 0.078 |  |
| >=3584 | Ref | 0.99 (0.97, 1.02) | 0.649 | 0.98 (0.96, 1.01) | 0.117 | 0.98 (0.95, 1.01) | 0.143 |  |
| Total time of PA (mins/week) |  |  |  |  |  |  |  | 0.523 |
| <600 | Ref | 0.99 (0.96, 1.03) | 0.722 | 0.97 (0.94, 1.00) | 0.067 | 0.97 (0.94, 0.99) | 0.013 |  |
| >=600 | Ref | 0.99 (0.97, 1.01) | 0.357 | 1.00 (0.98, 1.02) | 0.792 | 1.00 (0.97, 1.03) | 0.931 |  |
| Smoking status |  |  |  |  |  |  |  | 0.275 |
| Never | Ref | 0.99 (0.96, 1.01) | 0.304 | 0.99 (0.97, 1.01) | 0.192 | 0.98 (0.95, 1.00) | 0.083 |  |
| Former | Ref | 0.96 (0.92, 1.00) | 0.078 | 0.94 (0.90, 0.98) | 0.009 | 0.98 (0.94, 1.02) | 0.361 |  |
| Current |  | 1.04 (0.98, 1.09) | 0.172 | 1.01 (0.97, 1.05) | 0.638 | 0.99 (0.96, 1.03) | 0.63 |  |
| Alcohol Consumption | |  |  |  |  |  |  | 0.871 |
| Never | Ref | 0.95 (0.90, 1.01) | 0.097 | 0.97 (0.92, 1.03) | 0.356 | 1.00 (0.94, 1.06) | 0.996 |  |
| Former | Ref | 1.03 (0.97, 1.09) | 0.281 | 0.99 (0.94, 1.04) | 0.657 | 0.95 (0.91, 1.00) | 0.044 |  |
| Mild | Ref | 0.97 (0.92, 1.02) | 0.188 | 0.95 (0.91, 0.99) | 0.026 | 0.97 (0.92, 1.01) | 0.147 |  |
| Moderate | Ref | 1.02 (0.95, 1.10) | 0.587 | 1.02 (0.96, 1.09) | 0.495 | 1.04 (0.97, 1.11) | 0.305 |  |
| Heavy | Ref | 0.99 (0.94, 1.03) | 0.587 | 0.95 (0.92, 0.99) | 0.017 | 0.98 (0.94, 1.01) | 0.183 |  |
| Total score of HEI (2015 Edition) | |  |  |  |  |  |  | 0.567 |
| <51.44187 | Ref | 0.99 (0.96, 1.02) | 0.477 | 0.98 (0.96, 1.00) | 0.065 | 0.97 (0.94, 0.99) | 0.015 |  |
| >=51.44187 | Ref | 1.01 (0.98, 1.03) | 0.669 | 1.00 (0.98, 1.03) | 0.899 | 1.01 (0.98, 1.04) | 0.635 |  |
| Hypertension |  |  |  |  |  |  |  | 0.270 |
| No | Ref | 1.00 (0.98, 1.02) | 0.971 | 0.99 (0.98, 1.01) | 0.399 | 1.00 (0.98, 1.02) | 0.967 |  |
| Yes | Ref | 0.95 (0.90, 1.01) | 0.094 | 0.93 (0.89, 0.99) | 0.015 | 0.94 (0.89, 0.99) | 0.015 |  |
| Hyperlipidemia |  |  |  |  |  |  |  | 0.183 |
| No | Ref | 0.99 (0.98, 1.00) | 0.174 | 0.98 (0.97, 1.00) | 0.016 | 0.99 (0.97, 1.01) | 0.416 |  |
| Yes | Ref | 0.99 (0.96, 1.03) | 0.593 | 0.98 (0.95, 1.01) | 0.244 | 0.98 (0.95, 1.01) | 0.169 |  |

BMI: body mass index; HEI: healthy eating index; PA: physical activity.

**Supplementary Table S5.** ORs (95% CIs) of DM prevalence according to flavonoid intake in the NHANSE (2007-2010 and 2017-2018) excluding the population with impaired fasting glycaemia and impaired glucose tolerance.

| Flavonoid intake | Q1 | Q2 |  | Q3 |  | Q4 |  | OR (95%CI) | p for trend |
| --- | --- | --- | --- | --- | --- | --- | --- | --- | --- |
|  |  | OR (95%CI) | p Value | OR (95%CI) | p Value | OR (95%CI) | p Value |  |  |
| Total Sum of all 29 flavonoids (mg/day) | ≤20.645 | 20.645-49.735 |  | 49.735-140.145 |  | >140.145 |  |  |  |
| Crude Model | Ref | 0.79 (0.66, 0.94) | 0.01 | 0.90 (0.73, 1.09) | 0.267 | 1.04 (0.87, 1.25) | 0.641 | 1.03 (0.97, 1.10) | 0.286 |
| Model 1 | Ref | 0.79 (0.63, 0.99) | 0.039 | 0.76 (0.61, 0.96) | 0.025 | 0.76 (0.62, 0.93) | 0.01 | 0.93 (0.87, 0.99) | 0.023 |
| Model 2 | Ref | 0.76 (0.62, 0.95) | 0.015 | 0.74 (0.58, 0.94) | 0.016 | 0.76 (0.63, 0.93) | 0.01 | 0.93 (0.87, 1.00) | 0.043 |
| Model 3 | Ref | 0.78 (0.63, 0.97) | 0.025 | 0.76 (0.60, 0.97) | 0.03 | 0.80 (0.65, 0.98) | 0.029 | 0.94 (0.88, 1.01) | 0.104 |
| Total Flavan 3-ols (mg/day) | ≤4.605 | 4.605-13.245 |  | 13.245-59.525 |  | >59.525 |  |  |  |
| Crude Model | Ref | 0.76 (0.60, 0.95) | 0.017 | 0.62 (0.49, 0.79) | <0.001 | 1.02 (0.83, 1.25) | 0.878 | 1.00 (0.94, 1.08) | 0.892 |
| Model 1 | Ref | 0.80 (0.63, 1.02) | 0.074 | 0.65 (0.50, 0.84) | 0.002 | 0.81 (0.64, 1.02) | 0.068 | 0.93 (0.86, 1.01) | 0.07 |
| Model 2 | Ref | 0.78 (0.61, 1.00) | 0.052 | 0.64 (0.49, 0.83) | 0.001 | 0.81 (0.63, 1.02) | 0.075 | 0.94 (0.87, 1.01) | 0.109 |
| Model 3 | Ref | 0.79 (0.61, 1.01) | 0.058 | 0.65 (0.50, 0.84) | 0.002 | 0.84 (0.66, 1.07) | 0.154 | 0.95 (0.88, 1.03) | 0.224 |
| Subtotal Catechins (mg/day) | ≤4.505 | 4.505-12.745 |  | 12.745-37.725 |  | >37.725 |  |  |  |
| Crude Model | Ref | 0.80 (0.65, 0.98) | 0.036 | 0.66 (0.54, 0.81) | <0.001 | 0.97 (0.80, 1.18) | 0.757 | 0.99 (0.92, 1.05) | 0.683 |
| Model 1 | Ref | 0.83 (0.65, 1.06) | 0.123 | 0.70 (0.55, 0.88) | 0.004 | 0.81 (0.64, 1.02) | 0.069 | 0.93 (0.87, 1.01) | 0.069 |
| Model 2 | Ref | 0.81 (0.63, 1.03) | 0.082 | 0.69 (0.54, 0.88) | 0.004 | 0.80 (0.64, 1.01) | 0.065 | 0.94 (0.87, 1.01) | 0.087 |
| Model 3 | Ref | 0.82 (0.64, 1.04) | 0.097 | 0.70 (0.55, 0.89) | 0.005 | 0.83 (0.66, 1.05) | 0.124 | 0.95 (0.88, 1.02) | 0.165 |
| Total Isoflavones (mg/day) | ≤0.010 | 0.010-0.065 |  |  |  |  |  |  |  |
| Crude Model | Ref | 0.98 (0.86, 1.12) | 0.771 |  |  |  |  |  |  |
| Model 1 | Ref | 1.00 (0.86, 1.15) | 0.963 |  |  |  |  |  |  |
| Model 2 | Ref | 1.01 (0.86, 1.18) | 0.946 |  |  |  |  |  |  |
| Model 3 | Ref | 1.02 (0.87, 1.19) | 0.835 |  |  |  |  |  |  |
| Total Anthocyanidins (mg/day) | ≤ 0.125 | 0.125-1.910 |  | 1.910-9.340 |  | >9.340 |  |  |  |
| Crude Model | Ref | 0.88 (0.72, 1.09) | 0.25 | 1.07 (0.86, 1.34) | 0.538 | 0.93 (0.74, 1.16) | 0.51 | 0.99 (0.92, 1.07) | 0.865 |
| Model 1 | Ref | 0.85 (0.66, 1.10) | 0.202 | 0.96 (0.75, 1.24) | 0.757 | 0.77 (0.61, 0.98) | 0.033 | 0.93 (0.87, 1.01) | 0.084 |
| Model 2 | Ref | 0.83 (0.65, 1.07) | 0.147 | 0.95 (0.74, 1.23) | 0.694 | 0.78 (0.60, 1.00) | 0.046 | 0.94 (0.86, 1.02) | 0.124 |
| Model 3 | Ref | 0.86 (0.67, 1.11) | 0.24 | 0.99 (0.77, 1.27) | 0.927 | 0.82 (0.65, 1.05) | 0.11 | 0.95 (0.88, 1.04) | 0.251 |
| Total Flavanones (mg/day) | ≤ 0.020 | 0.020-0.660 |  | 0.660-17.730 |  | >17.730 |  |  |  |
| Crude Model | Ref | 1.23 (1.02, 1.48) | 0.029 | 0.90 (0.73, 1.11) | 0.322 | 1.01 (0.84, 1.23) | 0.878 | 0.97 (0.91, 1.03) | 0.316 |
| Model 1 | Ref | 1.09 (0.89, 1.33) | 0.387 | 0.89 (0.70, 1.13) | 0.339 | 0.85 (0.68, 1.07) | 0.156 | 0.93 (0.87, 1.00) | 0.05 |
| Model 2 | Ref | 1.09 (0.89, 1.34) | 0.393 | 0.91 (0.70, 1.18) | 0.465 | 0.86 (0.67, 1.10) | 0.219 | 0.93 (0.86, 1.02) | 0.109 |
| Model 3 | Ref | 1.11 (0.90, 1.36) | 0.323 | 0.95 (0.74, 1.23) | 0.7 | 0.87 (0.69, 1.11) | 0.257 | 0.94 (0.87, 1.02) | 0.142 |
| Total Flavones (mg/day) | ≤0.110 | 0.110-0.345 |  | 0.345-0.845 |  | >0.845 |  |  |  |
| Crude Model | Ref | 1.28 (1.08, 1.53) | 0.006 | 1.25 (1.01, 1.55) | 0.039 | 1.52 (1.26, 1.83) | <0.001 | 1.13 (1.06, 1.20) | <0.001 |
| Model 1 | Ref | 1.00 (0.82, 1.21) | 0.973 | 0.94 (0.74, 1.19) | 0.6 | 0.99 (0.84, 1.18) | 0.951 | 1.00 (0.94, 1.05) | 0.879 |
| Model 2 | Ref | 0.98 (0.80, 1.21) | 0.879 | 0.95 (0.73, 1.23) | 0.689 | 1.02 (0.83, 1.25) | 0.872 | 1.01 (0.93, 1.08) | 0.885 |
| Model 3 | Ref | 1.02 (0.83, 1.24) | 0.879 | 1.00 (0.77, 1.30) | 0.989 | 1.05 (0.86, 1.29) | 0.629 | 1.01 (0.94, 1.09) | 0.68 |
| Total Flavonols (mg/day) | ≤4.735 | 4.735-9.245 |  | 9.245-17.330 |  | >17.330 |  |  |  |
| Crude Model | Ref | 1.31 (1.11, 1.55) | 0.003 | 1.51 (1.27, 1.80) | <0.001 | 1.43 (1.19, 1.70) | <0.001 | 1.11 (1.05, 1.18) | 0.001 |
| Model 1 | Ref | 1.15 (0.88, 1.49) | 0.299 | 1.02 (0.83, 1.26) | 0.815 | 0.89 (0.72, 1.10) | 0.282 | 0.94 (0.88, 1.00) | 0.055 |
| Model 2 | Ref | 1.16 (0.88, 1.52) | 0.289 | 1.06 (0.85, 1.33) | 0.59 | 0.90 (0.71, 1.15) | 0.393 | 0.94 (0.88, 1.01) | 0.092 |
| Model 3 | Ref | 1.17 (0.89, 1.55) | 0.243 | 1.08 (0.86, 1.35) | 0.479 | 0.92 (0.73, 1.17) | 0.492 | 0.95 (0.88, 1.02) | 0.128 |

Crude model: unadjusted; Model 1: adjusted by age, race, BMI, and daily energy intake (kcal); Model 2: adjusted by age, race, BMI, and daily energy intake (kcal), total time of PA, smoking status, alcohol consumption, total score of HEI 2015 Edition, hypertension history, and hyperlipidemia history; Model 3: adjusted by age, sex, race, BMI, and daily energy intake (kcal), total time of PA, smoking status, alcohol consumption, total score of HEI 2015 Edition, hypertension history, and hyperlipidemia history. BMI: body mass index; HEI: healthy eating index; PA: physical activity.
